# Injuries among middle aged and older adult patients presenting to the emergency department: a retrospective cohort study

**DOI:** 10.3389/fragi.2025.1652588

**Published:** 2025-10-23

**Authors:** Abdullah M. Basnawi, Syeda Nafeesa Hashim, Sariya Khan, Husna Irfan Thalib, Ayesha Mirza Ayub, Sana Hashim, Fatimah Shakeel, Jumana Timraz, Fathma Islam

**Affiliations:** 1 Department of Emergency Medicine, University of Tabuk, Tabuk, Saudi Arabia; 2 General Medicine Practice Program, Batterjee Medical College, Jeddah, Saudi Arabia

**Keywords:** injuries, emergency department, older adults, middle age, >50 years, Saudi Arabia

## Abstract

**Introduction:**

As the global population ages, trauma among middle aged and older adults has become a significant public health concern, particularly in emergency care settings. In Saudi Arabia, the number of adults aged ≥50 years is steadily increasing, particularly in urban centers such as Jeddah, where multigenerational households and chronic health conditions influence injury patterns and healthcare utilization. Middle aged and older adults face higher rates of injury-related hospitalizations than younger populations, often exacerbated by physiological vulnerability and comorbid conditions. This retrospective study aimed to evaluate the sociodemographic factors, prevalent injury types, associated comorbidities, and clinical outcomes of middle aged and older adult’s patients presenting to the emergency department (ED) with trauma.

**Methods:**

A retrospective cohort study was conducted on patients aged ≥50 years who presented to the ED with trauma between January 2021 and December 2023. The assessed variables included sociodemographic data, injury severity, injury patterns, comorbidities, clinical management, and outcomes. Statistical analysis was performed using chi-square tests and logistic regression to identify the associations and predictors of surgical intervention.

**Results:**

A total of 248 middle aged and older adult patients with trauma were analyzed. Male patients sustained more severe injuries, with a statistically significant association between gender and injury severity (p = 0.028). No significant correlation was found between injury severity, age, and comorbidities. Logistic regression revealed that the mode of arrival and lower body injuries were significant predictors of surgical intervention (OR = 2.714, p = 0.046). Patients arriving by walk-in (OR = 7.560, p = 0.002) or personal vehicle (OR = 5.231, p = 0.006) were more likely to undergo surgery than those transported by ambulance. Surgical intervention was inversely associated with injury recurrence (OR = 0.214, p = 0.019), whereas the presence of comorbidities significantly increased the likelihood of surgical management (OR = 2.024, p = 0.031).

**Conclusion:**

Middle aged and Older adult trauma patients represent a complex and vulnerable population in emergency care. Male gender, lower limb injuries, comorbidities, and non-ambulance transport modes are significant predictors of surgical intervention. Identifying these factors can guide early triage, optimize care, and inform preventive strategies to improve outcomes and reduce the healthcare burden.

## 1 Introduction

Trauma in middle aged and older emergency patients is a relevant and growing problem in emergency medicine worldwide. With increasing life expectancy and a rising proportion of older individuals, emergency departments are increasingly confronted with older adult patients presenting diverse types of trauma. These injuries can lead to serious outcomes due to age-related physiological changes, multiple comorbidities, and impaired functional status (Rafaqat et al., 2024; Kara et al., 2014).

Middle aged and older adults, typically individuals 50 and above years of age, are particularly at risk of injury due to a combination of intrinsic and extrinsic factors. Physiological alterations, such as reduced bone density, impaired balance, delayed reaction time, and muscle wasting, significantly increase both the risk and severity of trauma. Moreover, comorbid conditions such as cardiovascular disease, diabetes, and cognitive impairment hinder recovery and predispose patients to complications. Consequently, even seemingly minor injuries, such as simple falls, can lead to fractures, head trauma, or substantial functional deterioration (Aleid et al., 2023).

In Saudi Arabia, the proportion of adults aged 50 years and older is increasing progressively, with projections that older adults will comprise up to 18% of the population by 2050 (Saquib et al., 2017). Jeddah, one of the largest urban regions in the Kingdom, has unique characteristics: many older adults live in multigenerational households, a source of social support, but may also influence patterns of injury and healthcare utilization (Almalki, 2021). Healthcare is provided by both public and private sectors, and older adults frequently have multiple chronic conditions that can potentially increase the complexity of trauma care (Saquib et al., 2017). These social, cultural, and healthcare considerations are key to understanding the patterns of injury and outcomes in this population (Bindawas et al., 2025).

Trauma in middle aged and older adult patients has implications far beyond the acute setting, most commonly resulting in hospitalization, prolonged length of stay, increased morbidity and mortality, and diminished independence and quality of life, usually necessitating long-term care or rehabilitation services (Aleid et al., 2023; Alzahrani, 2021). Repeated return visits to the emergency department due to injury-related problems are also common, constituting a significant burden on health systems (Aleid et al., 2023; Bindawas et al., 2025). In Saudi Arabia, functionally declining multimorbid older trauma patients create a high demand for inpatient and emergency services; hence, the requirement for appropriate preventive and management strategies (Harthi et al., 2024). A study at King Khalid University Hospital found that older adults had higher health spending due to chronic diseases (Boettiger et al., 2023). Nationally, the aging process is expected to account for a 63% increase in health expenditure related to non-communicable diseases by 2030 (Alshammari et al., 2019).

Early identification of risk factors and implementation of multidisciplinary management strategies are essential to prevent these adverse outcomes. Various studies have highlighted the severity of clinical implications; for example, an investigation in Konya, Turkey, revealed that 66% of older trauma patients required hospital admission, with femur fractures and intracranial injuries being significant contributors to mortality. Similarly, a study in Lebanon found that although most fall-related injuries were not immediately fatal, complications such as pneumonia, sepsis, and urinary tract infections resulted in a 2% mortality rate (Kara et al., 2014; Harthi et al., 2024). Unlike studies from Lebanon and Turkey, which reported higher inpatient admission rates and distinct injury mechanisms, our findings reflect patterns in Jeddah, where multigenerational housing, reliance on private transport, and lower ambulance use may shape trauma outcomes differently.

There is limited regional research in Saudi Arabia examining the impact of demographic and clinical variables on trauma outcomes among older adults, particularly with regarding specific injury patterns and comorbidities. Our research attempts to address this by investigating patients aged 50 years and older who presented to the emergency department to analyze the clinical and sociodemographic causes and outcomes of trauma. We aimed to identify significant risk factors, common types of injuries, and prognostic indicators to inform and optimize clinical management and prevention in the emergency context.

## 2 Materials and methods

### 2.1 Study design and setting

A retrospective cohort study was conducted between January 2021 to December 2023 to analyze the patterns of injury among older adult patients presenting to the Emergency Department (ED). This study aimed to evaluate the mechanisms of injury, associated risk factors, and outcomes of trauma in older adults. Data was collected over a 6-month period; from October 2024 to March 2025; using electronic medical records.

### 2.2 Study population and sampling technique

The study population comprised older adult patients (aged 50 years and above) who presented to the ED with an injury-related diagnosis. The inclusion criteria were complete documentation of the injury mechanism, medical history, and ED disposition. Patients younger than 50 years, those with nontraumatic conditions, and those with incomplete medical records were excluded. Patients were included using consecutive sampling to ensure adequate representation of various injury types and severities of the injuries.

### 2.3 Data collection and study tool

Patient data were extracted from hospital electronic medical records using a structured data abstraction form (Supplementary Appendix 1). The collected variables included demographic details (age, gender, and comorbidities), injury-related factors (mechanism of injury, anatomical site, and injury severity score), and ED outcomes (admission, discharge, or mortality). The mechanisms of injury were categorized as falls, road traffic accidents, burns, or other trauma-related incidents. Injury severity was assessed using the Injury Severity Score (ISS), derived from the Abbreviated Injury Scale (AIS) and Glasgow Coma Scale (GCS). Private transport was defined as arrival by personal vehicle, taxi, or walk-in (on foot). For analysis, ISS scores were classified as mild (1–8), moderate (9–15), and severe (>15), in accordance with standard definitions.

### 2.4 Ethical considerations

Ethical approval was obtained from the Institutional Review Board (IRB) of the International Medical Center, Jeddah (Approval number: [2024-11-256]). This study adhered to ethical guidelines, ensuring the confidentiality and anonymization of patient data. Informed consent was waived due to the retrospective nature of the study, according to institutional policies. All data were securely stored in password-protected Google Sheets and accessed only by authorized researchers.

### 2.5 Statistical analysis

Data was analyzed using SPSS version 30 (IBM, Chicago, IL, United States). Descriptive statistics, including frequencies and percentages, were used to summarize the patient’s characteristics and injury patterns. Chi-square tests were used to assess the association between injury mechanisms and demographic variables. Logistic regression analysis was performed to identify the predictors of severe injury and hospitalization, with results reported as adjusted odds ratios (AOR) and 95% confidence intervals (CI). Statistical significance was set at p < 0.05.

## 3 Results

A total of 248 older adult patients were included in this study. The participants were evenly distributed by gender, with 124 males (50%) and 124 females (50%). The age of the participants ranged from 50 to 99 years, with the highest proportion falling within the 60–69-year age group (31.8%), followed by those aged 70–79 years (30.2%) and 50–59 years (25%). Smaller proportions were noted among the 80–89 years (11.6%) and 90–99 years (1.2%) age categories. Regarding comorbidities, a significant majority (65%) of the participants reported having at least one comorbid condition, such as diabetes mellitus, hypertension, cardiovascular disease, or chronic kidney disease, while 35% reported no comorbidities. These baseline characteristics reflect a typical geriatric population vulnerable to injury and hospitalization, highlighting the importance of considering both age and comorbidity burden in clinical outcomes (Table 1).

**TABLE 1:** Socio-demographic characteristics of study participants.

Variable	Frequency (N)	Percentage (%)
Age		
50–59	62	25
60–69	79	31.8
70–79	75	30.2
80–89	29	11.6
90–99	3	1.2
Gender		
Males	124	50
Females	124	50
Comorbidities		
Yes	161	65
No	87	35

**FIGURE 1:**
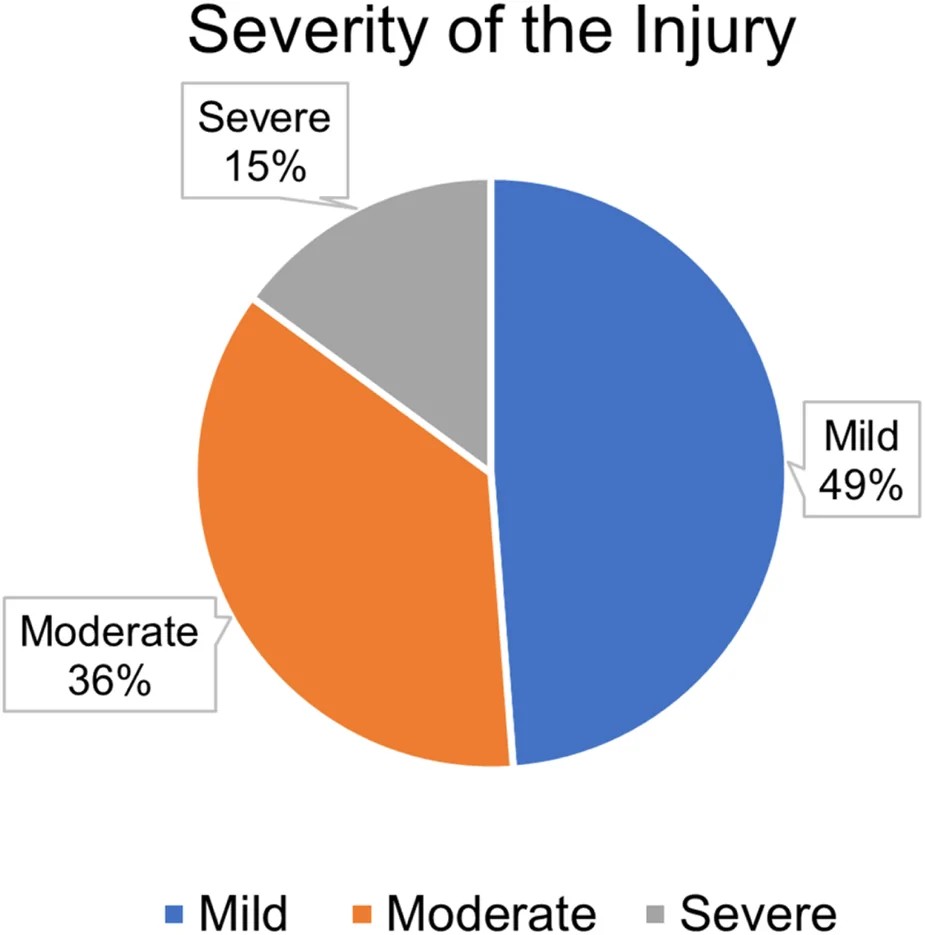
Participant distribution by injury severity classification.

**FIGURE 2:**
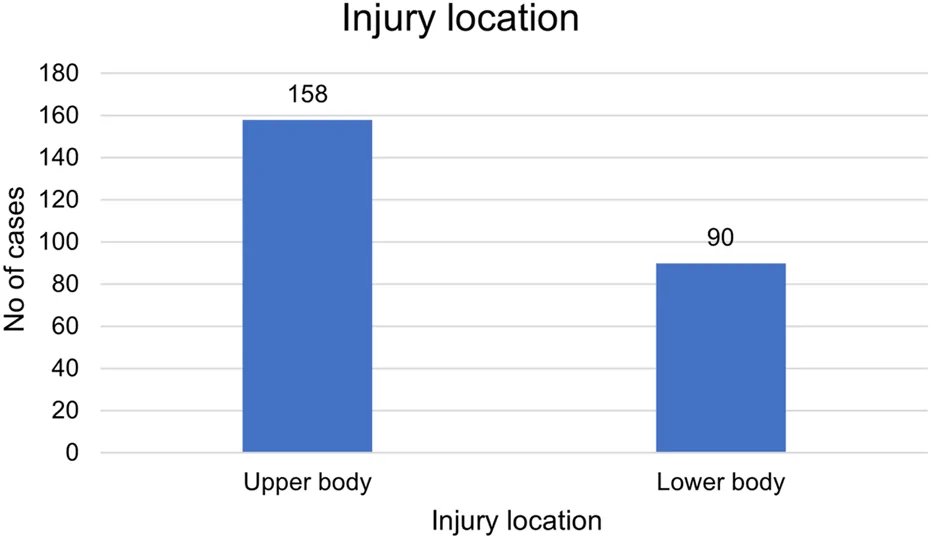
Participant distribution by injury location.

**FIGURE 3:**
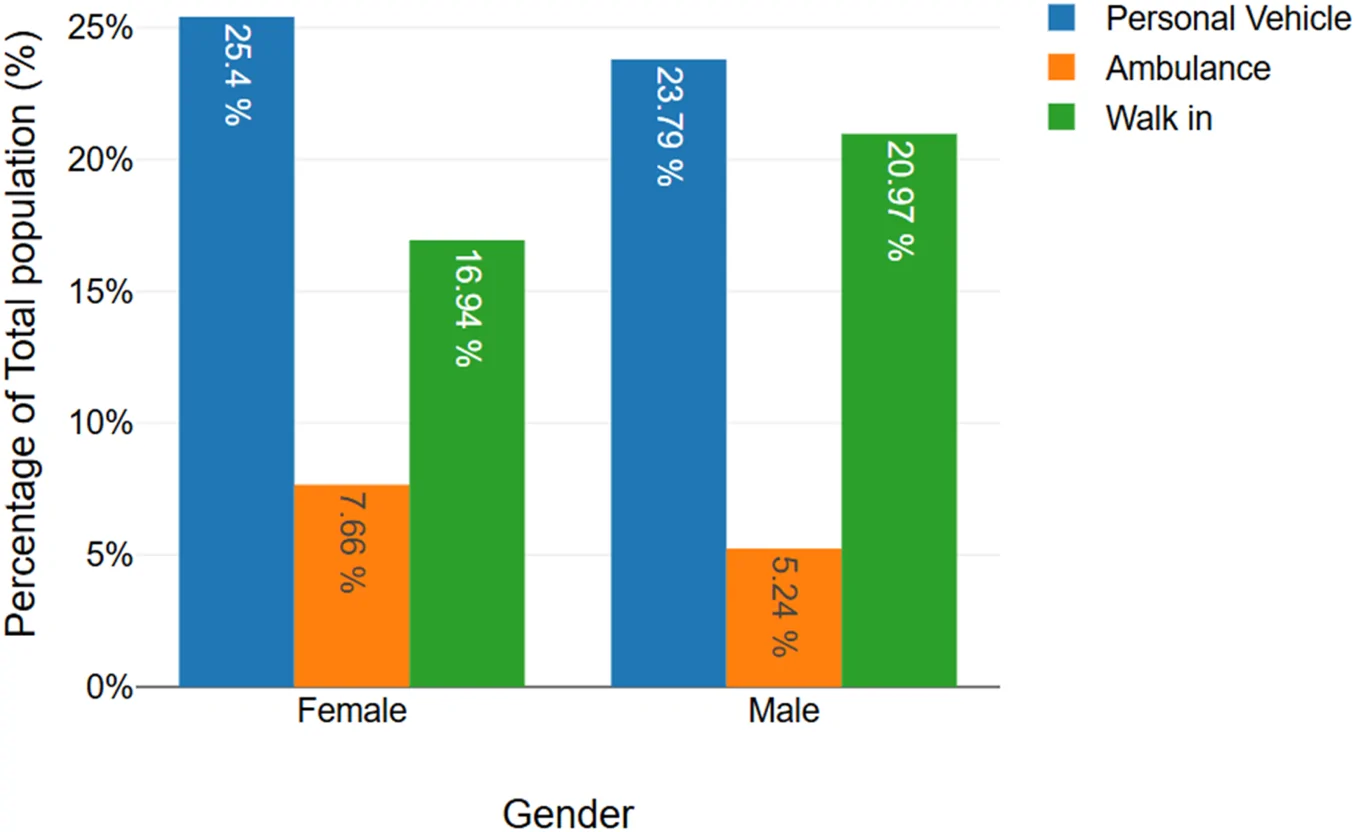
Chi-square analysis displayed through a bar graph showing association between gender and mode of arrival (p = 0.313).

Among the 248 older adult trauma patients included in the study, the distribution of injury severity, anatomical location, and mode of arrival revealed clinically significant trends. A total of 121 patients (48.8%) sustained mild injuries, 90 (36.3%) had moderate injuries, and 37 (14.9%) suffered severe trauma (Figure 1). Notably, severe injuries were more frequent among men, and this difference was statistically significant (p = 0.028). Upper limb injuries were the most common (Figure 2). Among the patients, 182 (73.4%) presented via personal vehicle or walk-in, compared with only 66 (26.6%) who arrived by ambulance. Chi-square analysis was performed to determine if there was any significance of gender to the mode of arrival, as depicted in Figure 3. This was found to be insignificant with a p value of 0.313 (Figure 3).

A chi-square analysis was conducted to evaluate the relationship between sociodemographic variables and the severity of the injuries sustained. Age was not significantly associated with injury severity (p = 0.817), indicating that the distribution of mild, moderate, and severe injuries was relatively similar across the different age groups. Interestingly, gender was significantly associated with severity (p = 0.028), with males experiencing a higher proportion of severe injuries than females. This finding suggests that gender may be a relevant factor when assessing injury risk among older adult patients. However, the presence of comorbidities did not show a significant association with injury severity (p = 0.987), suggesting that while comorbid conditions are common, they may not necessarily predispose individuals to more severe injuries in this population (Table 2).

**TABLE 2:** Chi-square analysis of socio-demographic factors influencing severity of injury.

Severity of the injury					
Variable		Mild	Moderate	Severe	p-value
Age	50–59	31	22	9	0.817
	60–69	42	25	12	
	70–79	35	30	10	
	80–89	13	11	5	
	90–99	0	2	1	
Gender	Males	56	42	26	0.028*
	Females	65	48	11	
Comorbidities	Yes	78	59	24	0.987
	No	43	31	13	

* Denotes significant p values < 0.05.

The necessity for surgical intervention among older adult participants was examined in relation to age, gender, and comorbidities. No statistically significant associations were found between age and surgery requirement (p = 0.227); or between gender and surgery requirement (p = 0.730). Similarly, the presence or absence of comorbidities did not significantly influence the likelihood of requiring surgery (p = 0.151). These findings suggest that the decision for surgical management in this cohort was not predominantly influenced by basic demographic characteristics or existing health conditions but likely depended on other clinical factors, such as the type and extent of injury sustained (Table 3).

**TABLE 3:** Chi-square analysis of socio-demographic factors influencing surgery required.

Surgery required				
Variable		Yes	No	p-value
Age	50–59	56	6	0.227
	60–69	65	14	
	70–79	64	11	
	80–89	21	8	
	90–99	2	1	
Gender	Males	105	19	0.730
	Females	103	21	
Comorbidities	Yes	139	22	0.151
	No	69	18	

* Denotes significant p values <0.05.

Further analysis was performed to explore the impact of sociodemographic factors on a range of outcomes, including injury location, injury recurrence, mode of arrival at the hospital, hospital admission requirements, and discharge destination. Age, gender, and comorbidities were not significantly associated with injury location, injury recurrence, mode of arrival, or the need for hospital admission. However, significant associations were found between age and discharge destination (p = 0.000), between comorbidities and discharge destination (p = 0.007). These findings suggest that older age and the presence of comorbidities influence whether patients are discharged home, transferred to another facility, or experience other discharge outcomes, emphasizing the importance of these variables in planning post-hospital care and rehabilitation strategies for older adult patients (Table 4).

**TABLE 4:** Chi-square analysis of socio-demographic factors influencing various outcomes.

Variable	Age	Gender	Comorbidities
Injury Location	0.774	0.428	0.206
Injury Recurrence	0.248	0.129	0.650
Mode of Arrival	0.305	0.313	0.081
Hospital Admission Required	0.182	0.321	0.212
Discharged to	0.000*	0.338	0.007*

* Denotes significant p values <0.05.

Table 5 shows the results of a binary logistic model estimating the predictors of the likelihood of requiring surgery among older adult patients. Age had increasing odds with increasing age, particularly in the 90–99 years category (OR = 28.791); however, this was not statistically significant (p = 0.066). Gender was not a significant influence on outcomes from surgery (OR = 1.540, p = 0.387), nor were comorbidities (OR = 0.245, p = 0.10). Injury severity was positively correlated, with severe injury correlating more strongly, nearly at significance, with the need for surgery (OR = 3.691, p = 0.052), than to mild injury.

**TABLE 5:** Binary logistics regression: Effect of variables on surgery required.

Variable		OR	CI	P-value
Age	50–59	1		
	60–69	3.040	0.817–11.315	0.097
	70–79	1.707	0.432–6.752	0.446
	80–89	2.986	0.591–15.089	0.186
	90–99	28.791	0.083–0.719	0.066
Gender	Males	1		
	Females	1.540	0.579–4.099	0.387
Comorbidities	Absent	1		
	Present	0.245	0.579–4.099	0.10
Severity	Mild	1		
	Moderate	2.447	0.804–7.446	0.115
	Severe	3.691	0.990–13.765	0.052
Injury Location	Upper Body	1		
	Lower Body	2.714	1.019–7.223	0.046*
Injury Recurrence	No	1		
	Yes	0.369	0.369–0.081	0.199
	Prior	0.607	0.074–4.967	0.641
Mode of Arrival	Ambulance	1		
	Personal vehicle	0.858	0.207–3.550	0.833
	Walk-In	0.372	0.068–2.042	0.255

* Denotes significant p values < 0.05.

A statistically significant correlation was found with injury location; patients with lower body injuries were more likely to undergo surgery than those with upper body injuries (OR = 2.714, p = 0.046). Injury recurrence and mode of arrival (ambulance, personal vehicle, or walk-in) were not predictive of the need for surgery. These findings suggest that while age and injury severity have strong trends, the injury location is a significant predictor of surgical selection in this population.

Table 6 shows the logistic model analyzing the determinants of the need for surgery. Unlike the first model, comorbidities were significantly associated with increased odds of surgery (OR = 2.024, p = 0.031), highlighting the clinical significance of comorbidities in the surgical planning. Gender and age were not significant predictors in this model, but females had marginally elevated, though non-significant, odds (OR = 1.444, p = 0.219).

**TABLE 6:** Binary logistics regression: Effect of variables on injury location required.

Variable		OR	CI	P-value
Age	50–59	1		
	60–69	0.775	0.364–1.648	0.507
	70–79	0.673	0.314–1.441	0.308
	80–89	0.458	0.158–1.330	0.151
	90–99	0.000	0.000-	0.999
Gender	Males	1		
	Females	1.444	0.804–2.595	0.219
Comorbidities	Absent	1		
	Present	2.024	1.067–3.836	0.031*
Severity	Mild	1		
	Moderate	1.138	0.611–0.139	0.683
	Severe	1.097	0.440–2.736	0.843
Surgery Required	No	1		
	Yes	3.121	1.218–0.000	0.018*
Injury Recurrence	No	1		
	Yes	0.214	0.059–0.775	0.019*
	Prior	0.611	0.139–2.681	0.514
Mode of Arrival	Ambulance	1		
	Personal vehicle	5.231	1.604–17.061	0.006*
	Walk-In	7.560	2.152–26.554	0.002*

* Denotes significant p values <0.05.

Injury severity was not statistically significant, although recurrent and severe injuries were more probable than mild injuries. Remarkably, recurrence of injury was significantly negatively correlated with surgery (OR = 0.214, p = 0.019), suggesting that recurrent injuries may not always require operative treatment. Patients who eventually required surgery were significantly associated with increased odds (OR = 3.121, p = 0.018), which again points to its clinical value in outcome modeling.

One of the surprising results was the strong influence of the mode of arrival. Patients arriving in a personal vehicle (OR = 5.231, p = 0.006) or walking in (OR = 7.560, p = 0.002) were more likely to have undergone surgery than those arriving by ambulance. The results emphasize the importance of logistics and clinical presentation factors in surgical decision-making.

## 4 Discussion

This retrospective study aimed to investigate the outcomes of injuries and surgical interventions required for the older adult population attending a healthcare facility in Jeddah, Saudi Arabia. The range of patient age groups varied from 55 to 99 years, highlighting the influence of pre-existing conditions as well as the location of injuries resulting in the clinical attendance of older adult patients to the ER.

The findings of this study both agree and disagree with the literature on many occasions. Age was not significantly associated with injury severity or the need for surgery, which contrasts with the overall literature that unanimously presents older age as an independent risk factor for greater injury severity and greater need for surgery. However, age was significantly and strongly related to the discharge destination. From the literature, while age is not commonly used for early clinical decision-making, it becomes a problem when planning post-acute care and discharge destinations, probably because older patients require rehabilitation (Pagin et al., 2020; Klukowska-Rötzler et al., 2025).

However, gender was significantly linked to the severity of injury, with more severe injuries in men. This finding is consistent with other studies that have shown a heightened risk and severity of injury in men, particularly older men, from occupational exposures or unequal physical imbalances. Surgical consideration was not influenced by gender, consistent with the broad opinion that surgical intervention is largely based on clinical presentation and not demographic variables (Klukowska-Rötzler et al., 2025; Gürpinar et al., 2024).

Comorbidities were also significant predictors of both surgical treatment and discharge location. This indicates that although comorbidities themselves are not the main driving force for making decisions at first sight, they do influence treatment (Gürpinar et al., 2024).

The least expected finding was the arrival mode. Contrary to triage expectations, individuals who presented on foot or by personal vehicle were more likely to undergo surgery than those who arrived by ambulance. This is contrary to the typical literature, which generally expects ambulatory arrivals to equate to increased acuity and increased requirement for surgery (Gürpinar et al., 2024).

Our study provides valuable insights into injuries among older patients presenting to the ER of a single healthcare center in Jeddah, Saudi Arabia; however, it is essential to acknowledge its strengths and limitations. One of the main strengths of our study is the examination of a wide range of demographic, clinical, and outcome-related variables, including comorbidities, injury severity, mode of arrival, surgical intervention, and discharge disposition, which allows for a deeper understanding of the complexities of geriatric trauma. Subgroup analyses further supplemented the findings by highlighting significant associations, such as those between gender and injury severity and as well as comorbidities and discharge outcomes. Furthermore, the balanced representation of male and female patients, combined with a relatively large sample size, improved the internal validity of the results. Given Jeddah’s urban environment and reliance on family based caregiving, these contextual factors may influence the patterns of transport and clinical outcomes in older patients with trauma. This study also addresses a significant gap in the regional literature by focusing on an understudied yet vulnerable population. It highlights key trends, such as the prevalence of falls, consistent with international findings, and provides valuable insights for improving emergency care.

Despite these strong findings, our study had certain important limitations. As a retrospective study, it is intrinsically dependent on the reliability and completeness of existing digital medical data, which may result in information bias due to missing or inconsistent data.

One major limitation is the absence of several important variables in the dataset. For instance, the mechanism of injury of how the patient was injured (e.g., falls, burns, road traffic accidents- RTA) was not documented in the hospital’s electronic records, preventing us analyzing the injury patterns in detail. Similarly, data regarding the presence or absence of comorbidities and details of each individual comorbid condition (e.g., hypertension and diabetes mellitus) were not systematically recorded in the hospital database, limiting our ability to assess their possible impact on patient outcomes. Additionally, information regarding the type of surgical intervention (e.g., orthopedic, neurosurgical, or other specialties) and the incidence of fracture related specifically to falls was unavailable due to inadequate reporting of injury mechanisms. We were unable to analyze the length of hospital stay, as this variable was not consistently documented in the medical records. Lastly, other potential confounding variables, such as the patient’s living arrangements and socioeconomic background, were not included due to their unavailability in the hospital records, limiting our ability to assess their potential influence on injury risk and recovery results.

The lack of these variables largely arises from limitations in the hospital’s prior electronic medical record (EMR) system, where comprehensive injury-related and comorbidity-specific data were either inconsistently documented or entirely omitted from the records.

Furthermore, our data were drawn from a single-center design in Jeddah, the western region of Saudi Arabia. Therefore, this limits the generalizability of our findings to other healthcare settings and different parts of the country, where demographic characteristics and healthcare practices may differ.

The results of this cohort have important clinical implications for the management of older adult trauma patients. The post-hospital care management for these trauma patients suggests that clinicians should account for higher healthcare utilization by necessitating proactive planning through a multidisciplinary approach, incorporating an emphasis on rehabilitation strategies and social support for better care and outcomes. The patterns of mode of arrival for older adult patients function as both logistical and clinical presentations, making it a pivotal factor in surgical intervention. Patients arriving via personal vehicle and walk-in often presented with greater injuries, highlighting the prerequisite for accelerated surgical triage and preparedness. For older adults, recognizing this association enables healthcare professionals to improve the efficiency of injury assessment protocol for this age group thereby enhancing the quality of highly coordinated hospital care and providing ultimate patient safety.

## 5 Future recommendations

As the population of the world continues to age, older adult patients with injuries are presenting increasingly unique challenges to emergency departments. They do so in addition to chronic illness, polypharmacy, and atypical symptoms such as those described above (Carpenter et al., 2015). This highlights the necessity for us to shift our emergency care systems and pay greater attention to the singular needs of older individuals.

In the future, more than simply measuring the degree of injury should be undertaken. Frailty and functional status should be monitored on a daily basis, which could give a better prediction for this population and enable clinicians to make the best decisions (Joseph et al., 2014). Prevention community programs, such as home adjustment, fall prevention, and early rehabilitation, also have the potential to reduce hospitalization and improve long-term recovery (Gillespie et al., 2012). Implementing fall-prevention programs, early referral to physical therapy, and community awareness on timely ambulance activation may reduce recurrent injuries.

While this is valuable information based on a retrospective review, several healthcare center prospective studies need to be conducted to confirm findings and determine evidence-based procedures for geriatric patients.

## 6 Conclusion

This study identifies key factors influencing surgical intervention in older adult trauma patients, notably male gender, lower extremity injuries, and comorbidities. The mode of arrival significantly predicts the need for surgery, with non-ambulance arrivals associated with higher operative rates, suggesting potential gaps in prehospital care. Surgical treatment is linked to reduced injury recurrence, highlighting the importance of timely intervention. Given that falls remain the predominant cause of injury, focused prevention strategies are critical. These findings highlight the necessity for tailored emergency protocols that integrate patient demographics and injury patterns to optimize outcomes in older adults. Further research is essential to validate these predictors and improve long-term recovery in this growing population.

## Data Availability

The original contributions presented in the study are included in the article/Supplementary Material, further inquiries can be directed to the corresponding author.
