# Correction: Injuries among middle aged and older adult patients presenting to the emergency department: a retrospective cohort study

**DOI:** 10.3389/fragi.2026.2034717

**Published:** 2026-10-30

**Authors:** Abdullah M. Basnawi, Syeda Nafeesa Hashim, Sariya Khan, Husna Irfan Thalib, Ayesha Mirza Ayub, Sana Hashim, Fatimah Shakeel, Jumana Timraz, Fathma Islam

**Affiliations:** 1 Department of Emergency Medicine, University of Tabuk, Tabuk, Saudi Arabia; 2 General Medicine Practice Program, Batterjee Medical College, Jeddah, Saudi Arabia

**Keywords:** injuries, emergency department, older adults, middle age, >50 years, Saudi Arabia

Affiliation “Department of Emergency Medicine, International Medical Center, Jeddah, Saudi Arabia” was erroneously included in the paper. This affiliation has now been removed for author Abdullah M. Basnawi.

The original version of this article has been updated.

