# Supplementary material for: Injuries among middle aged and older adult patients presenting to the emergency department: a retrospective cohort study
Source: Front Aging. 2025 Oct 23;6:1652588. doi: 10.3389/fragi.2025.1652588 (PMC12589095; doi:10.3389/fragi.2025.1652588)
Supplement: Supplementary file 1 [file Table1.docx]

**Supplementary Material**

**Supplementary Appendix 1**

Demographic variables

1. Age

2. Gender (Male/Female)

3. Comorbidities (DM, HTN, etc)

Injury Variables

1. Type of injury (fractures, sprains, lacerations, contusions, etc.)

2. Injury severity (mild, moderate, severe)

3. Injury mechanism

4. Injury location

5. Injury recurrence if any prior injuries

Clinical variables

1. Time of injury (morning/evening)

2. Arrival time to ER (time between injury occurrence and arrival to ER)

3. Mode of arrival (ambulance, private transport, or walk-in)

4. Initial Glasgow

5. What medical treatment was given?

6. Did they require surgery?

Outcomes

1. Length of stay in ER

2. Hospital admission required; if so, how many days?

3. Discharge details (home, another facility, or dead)
